# Enhanced Performance of Magnetic Graphene Oxide-Immobilized Laccase and Its Application for the Decolorization of Dyes

**DOI:** 10.3390/molecules22020221

**Published:** 2017-02-01

**Authors:** Jing Chen, Juan Leng, Xiai Yang, Liping Liao, Liangliang Liu, Aiping Xiao

**Affiliations:** Institute of Bast Fiber Crops, Chinese Academy of Agricultural Sciences, Changsha 410205, China; chenjingcaas@yahoo.com (J.C.); juanlengcaas@yahoo.com (J.L.); xiaiyang@yahoo.com (X.Y.); lipingliaocaas@yahoo.com (L.L.)

**Keywords:** dyes, graphene oxide, laccase, magnetic nanoparticles

## Abstract

In this study, magnetic graphene oxide (MGO) nanomaterials were synthesized based on covalent binding of amino Fe_3_O_4_ nanoparticles onto the graphene oxide (GO), and the prepared MGO was successfully applied as support for the immobilization of laccase. The MGO-laccase was characterized by transmission electron microscopy (TEM) and a vibrating sample magnetometer (VSM). Compared with free laccase, the MGO-laccase exhibited better pH and thermal stabilities. The optimum pH and temperature were confirmed as pH 3.0 and 35 °C. Moreover, the MGO-laccase exhibited sufficient magnetic response and satisfied reusability after being retained by magnetic separation. The MGO-laccase maintained 59.8% activity after ten uses. MGO-laccase were finally utilized in the decolorization of dye solutions and the decolorization rate of crystal violet (CV), malachite green (MG), and brilliant green (BG) reached 94.7% of CV, 95.6% of MG, and 91.4% of BG respectively. The experimental results indicated the MGO-laccase nanomaterials had a good catalysis ability to decolorize dyes in aqueous solution. Compared with the free enzyme, the employment of MGO as enzyme immobilization support could efficiently enhance the availability and facilitate the application of laccase.

## 1. Introduction

Enzymes are biological catalysts with excellent catalysis properties in various fields [1]. Because of the high activity and specificity, enzymes are widely used in industrial biosynthesis and environmental protection [2]. However, enzymes are expensive, unstable in complicated solution environments, and difficult to separate from solution. Enzyme immobilization could effectively prolong the activity, improve the stability, and provide the reusability of enzyme [3,4]. Immobilized enzymes also showed higher pH and temperature endurance range, satisfied stability, and simple product purification [5,6].

Various inorganic and organic nanomaterials such as silica nanoparticles, carbon nanotubes, gold nanoparticles, and metal oxide nanoparticles have been used as carriers to immobilize enzymes [7,8,9,10,11]. Among them, magnetic nanomaterials show many advantages including large surface areas for high immobilization amount of enzymes, facile modification, simple separation with a magnet, and high reusability [12]. Graphene oxide (GO) has also attracted the attention of researchers due to its typical large surface area, good biocompatibility, excellent stability, and high adsorption capacity. GO contains carboxyl, hydroxyl, and epoxy groups and was generally applied in drug delivery, sensors, and nanocomposites [13]. The combination of magnetic nanomaterials and GO remain an advantage of these two materials. The magnetic response property, large surface area, two-dimensional structure, easy surface modification, large enzyme immobilization capacity, simple preparation, and satisfactory reusability of magnetic GO (MGO) has received considerable attention in enzyme immobilization and related research [14,15]. MGO and graphene-based magnetic nanocomposites were synthesized as enzyme immobilization support for catalase, glucoamylase, lipase, and β-galactosidase [15,16,17,18,19]. The synthesized immobilized enzyme showed high activity recovery, easy recycling, improved catalytic activity, and stability compared to the free enzyme. However, the immobilization of laccase on MGO has seldom been reported.

Water pollution has been a universal crisis in modern industry over the years. Especially, dye wastewater is one of the most serious problems because of the many kinds of dyes used in the textile, paper, and food industries. Most dyes and pigments are toxic, complex, have low biodegradability, and are carcinogenic. They have been shown to cause much damage due to the resistance to degradation and the perilous affect to plants, aquatic organisms, and human beings [20]. Triphenylmethane dyes consisting of crystal violet (CV) and malachite green (MG) and azo dyes such as brilliant green (BG) were all widely used in various industries. Dye wastewater can be treated by conventional physical and chemical methods including adsorption, coagulation, and oxidation [21,22,23]. Among them, dye decolorization using enzymes such as laccase has gained great attention due to its processing efficiency [24,25].

Laccase (EC 1.10.3.2) is a copper-containing oxidase widely distributed in plants, insects, and fungi [26]. Laccase mainly catalyze the polymerization or depolymerization processes of lignin formation in plants. It is widely used in the textile, dyeing, and printing industries for the decolorization of dyes and pulp and the delignification of woody biomass [27]. In addition, laccase can be applied to the oxidation of various phenolic and non-phenolic compounds including various dyestuffs and environmental pollutants [28]. Based on previous reports, laccase has received attention for the treatment of dyes and phenolic compounds. Dye decolorization using immobilized laccase has also been reported in recent years [24,29]. Therefore, using laccase and immobilized laccase as a biocatalyst for the decolorization of dyes is a promising prospect.

Enzyme immobilization methods include entrapment, cross-linking, physical adsorption, and covalent binding to supports. Among these, covalent binding has shown advantages in forming stable binding between enzymes and supports [30]. Bifunctional cross-linking agents such as glutaraldehyde, maleic anhydride, and genipin are important in the immobilization of enzymes [31]. In this study, MGO-immobilized laccase (MGO-laccase) was synthesized and applied in the decolorization of CV, MG, and BG. Laccase and animo Fe_3_O_4_ nanoparticles were covalently bound on GO after the activation of GO using 1-ethyl-3-(3-dimethylaminopropyl) carbodiimide (EDC) and N-hydroxy sulfosuccinimide (NHS) as the bifunctional cross-linking agents. The prepared MGO and MGO-laccase were characterized by transmission electron microscopy (TEM) and a vibration sample magnetometer (VSM). In order to obtain the optimum reaction condition, the activities of free laccase and MGO-laccase at different pH and temperature conditions were measured and compared. At the optimum condition, highly efficient decolorization of CV, MG, and BG was achieved by MGO-laccase.

## 2. Results and Discussion

### 2.1. Characterizations of MGO-Laccase

**Figure 1:**
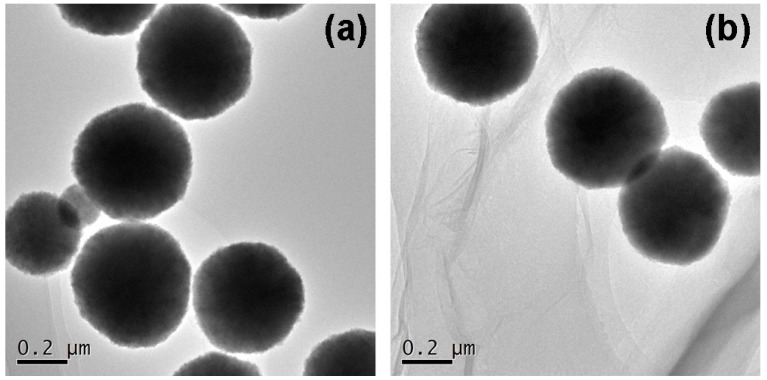
The TEM images of (**a**) amino Fe_3_O_4_ nanoparticles and (**b**) MGO-laccase.

Figure 1 shows the TEM characterization images of amino Fe_3_O_4_ nanoparticles and MGO-laccase. The animo Fe_3_O_4_ nanoparticles presented a round shape, and the average diameter is about 200 nm in Figure 1a. The shape and size of amino Fe_3_O_4_ nanoparticles are in accord with other reported values [32]. After the combination of GO, it can be clearly seen in Figure 1b that amino Fe_3_O_4_ nanoparticles steadily bound with GO. The sheet structure with a wrinkled edge of GO has been seen previously [33].

**Figure 2:**
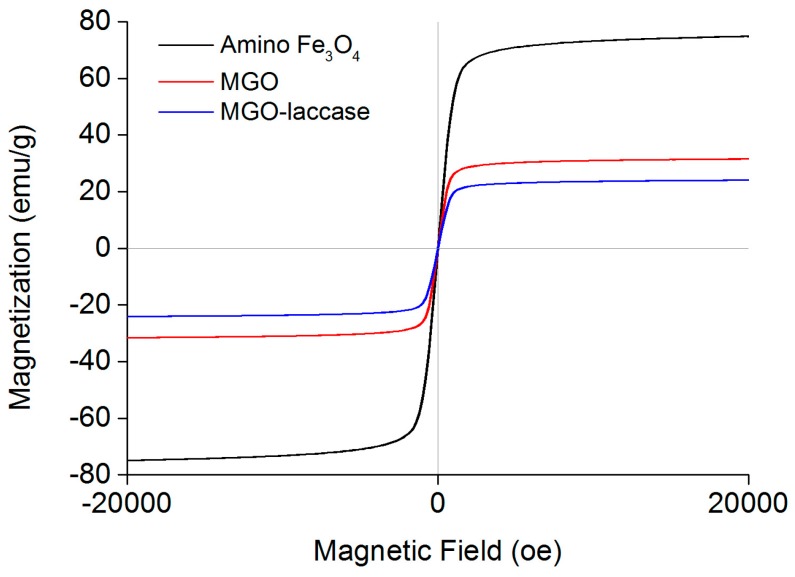
The magnetization curves of amino Fe_3_O_4_ nanoparticles (**black**), MGO (**red**), and MGO-laccase (**blue**).

The magnetic properties of amino Fe_3_O_4_ nanoparticles, MGO, and MGO-laccase were investigated using a VSM at room temperature, and their magnetization curves are shown in Figure 2. The maximum saturation magnetizations of MGO-laccase were 24.0 emu/g, which was lower than that of amino Fe_3_O_4_ nanoparticles (74.9 emu/g) and MGO (31.5 emu/g). The lower maximum saturation magnetizations of MGO-laccase was due to the increasing amount of nonmagnetic GO and laccase on the surface after functionalization and the relatively reduced amount of Fe_3_O_4_ nanoparticles [34,35]. Although the maximum saturation magnetizations of MGO-laccase declined to a certain extent, it still had an accepted magnetic response for rapid separation from the reaction medium [36].

### 2.2. Immobilization of Laccase on MGO-Laccase

**Figure 3:**
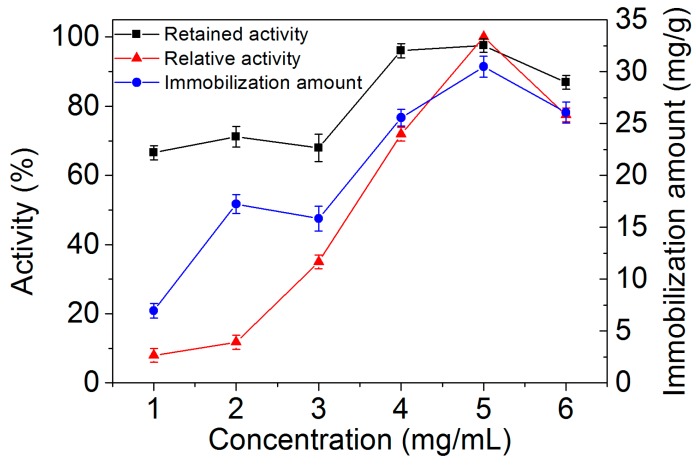
Immobilization amounts of laccase, retained activities of immobilized laccase on MGO-laccase, and relative activities of MGO-laccase at different laccase concentrations (1.0–6.0 mg/mL).

The concentration of laccase affected the immobilization, and it needed to be optimized to obtain the maximum amount of retained activity and enzyme immobilization. Therefore, the immobilization amounts of laccase on MGO, the retained activities of MGO-laccase, and the relative activities after immobilization were all investigated. As shown in Figure 3, the immobilization amount of laccase and the relative activity of MGO-laccase increased with the increase in laccase concentration and reached the highest (the immobilization amount of laccase was 30.0 mg/g and the relative activity of MGO-laccase was 97.9%) when the concentration of laccase was 5.0 mg/mL. Meanwhile, the retained activities of MGO-laccase reached 96% and 97.9% when the concentration of laccase was 4.0 and 5.0 mg/mL, respectively. However, a reduction of data was observed when the concentration of laccase was more than 5.0 mg/mL. This might be because excessive enzymes blocked the intermolecular space and restrained the transmission of substrate [37]. As a result, the optimum concentration of laccase was set at 5.0 mg/mL.

### 2.3. Effect of pH on the Activities of Free Laccase and MGO-Laccase

**Figure 4:**
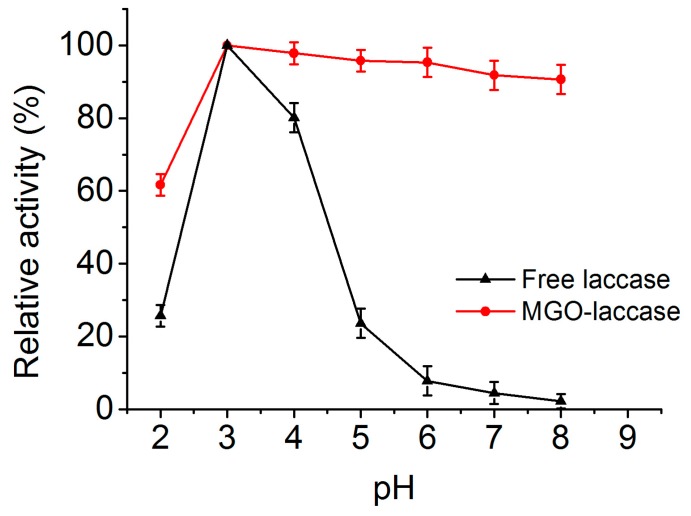
Effect of pH on the activities of free laccase (**black**) and MGO-laccase (**red**).

The effect of pH on the activities of free laccase and MGO-laccase were investigated at room temperature in the pH range 2.0–8.0, and the results are shown in Figure 4. The highest activity of the free laccase and MGO-laccase was individually considered as 100% and the activities at the other pH were noted proportional to the corresponding highest values. As shown in Figure 4, free laccase and MGO-laccase exhibited the maximum activity at pH 3.0. When pH was higher than 3.0, the activity of free laccase decreased sharply, while MGO-laccase showed relatively higher activity compared to that of free laccase. As reported, the stability of fungal laccases is generally higher at acidic pH. Diao and coauthors mentioned that the optimal pH for the activity of immobilized *Panus conchatus* laccase is 3.2 [38]. The improved pH tolerance of MGO-laccase against pH changes in solution is attributed to the buffer function of GO. In an acidic medium, the hydrogen ions are attracted and consumed by the negatively charged GO, which is helpful in preventing the hydrogen ions from contacting with the enzymes [39,40]. The improved performance might also be due to the conformational changes resulting in a suitable open conformation, with few restrictions to substrates produced by immobilization. These result is in agreement with other studies [41].

### 2.4. Effect of Tempertature on the Activities of Free Laccase and MGO-Laccase

**Figure 5:**
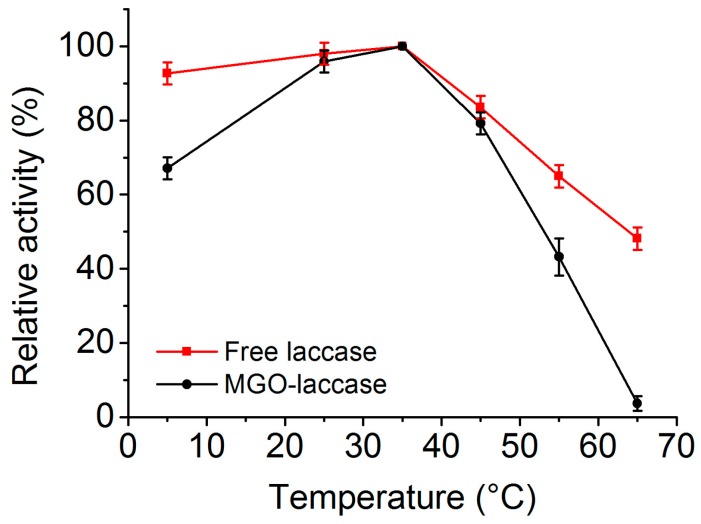
Effect of temperature on the activities of free laccase (**black**) and MGO-laccase (**red**).

The effect of temperature on the activities of free laccase and MGO-laccase was assayed at pH 3.0 and different temperatures in a range of 5–65 °C. The highest activity of the free laccase and MGO-laccase was individually considered as 100%, and the activities at the other temperatures were noted to be proportional to the corresponding highest values. As seen in Figure 5, the highest activities of free laccase and MGO-laccase both occurred at 35 °C. When the temperature increased from 35 to 65 °C, the activities of free laccase and MGO-laccase decreased because of the enzyme deactivation at relative high temperatures. However, MGO-laccase showed relatively higher activity compared with that of free laccase at high temperature, which means less sensitivity to temperature and more rigidity in conformational changes. This result was probably due to the strength of interactions between enzyme and matrix or a low restriction in the substrate diffusion [42].

### 2.5. Reusability of MGO-Laccase

**Figure 6:**
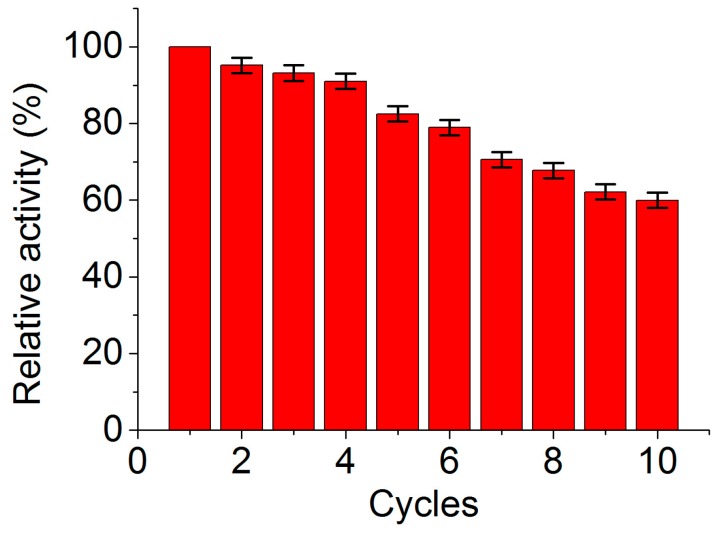
Reusability of MGO-laccase over ten consecutive cycles.

The reusability of immobilized enzyme was an important factor for practical applications to make the process economic and feasible. The activity of MGO-laccase remained 59.8% even after ten consecutive cycles of reuse (Figure 6). This result indicates that the MGO-laccase exhibits appropriate reusability in consecutive cycles of reuse. The reduction in residual activity over the ten cycles might be because of the aggregation of nanomaterials, the enzyme leakage from the supporters, and the deactivation of enzyme [9,43]. Considering the simple and rapid separation from reaction solution by an ordinary magnet, the MGO-laccase could be reused repeatedly in order to reduce the cost and simplify the processes.

### 2.6. Decolorization of Dyes

**Figure 7:**
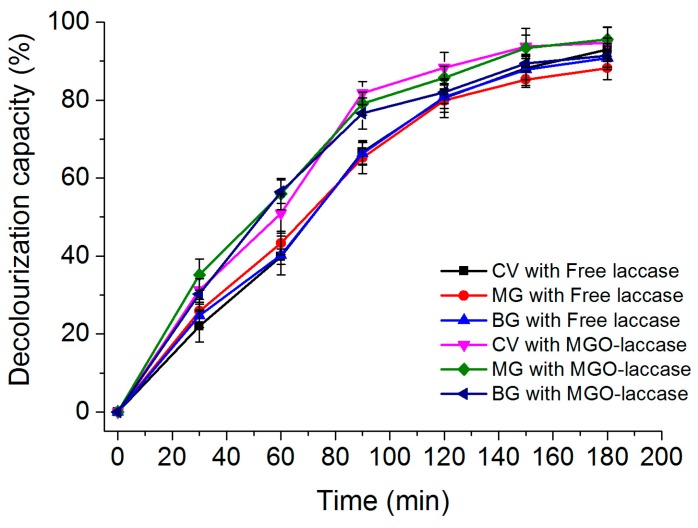
The removal rates of dyes (CV, MG, and BG) with free laccase and MGO-laccase.

The decolorization capacities of various dyes including CV, MG, and BG using free laccase and MGO-laccase were both assessed. As shown in Figure 7, 93.0% of CV, 88.2% of MG, and 90.8% of BG was respectively decolorized after 180 min incubation with free laccase in the optimum conditions. Meanwhile, 94.7% of CV, 95.6% of MG, and 91.4% of BG was respectively decolorized with MGO-laccase after 180 min of incubation. However, as a comparison, the decolorization ability of MGO was tested, and the result showed that only 13.2% of dyes decolorized after 180 min of incubation was reached. In general, the decolorization capacities of free laccase were equal to or even lower than that of the immobilized laccase. Obviously, MGO-laccase exhibited good decolorization capacity to dye solutions in this work. Combined with the appropriate reusability in consecutive cycles of reuse, the MGO-laccase is suitable as material efficient in the decolorization of dyes from aqueous solutions.

## 3. Materials and Methods

### 3.1. Materials

Laccase from *Trametes versicolor*, 2,2-azinobis-3-ethylbenzothiazoline-6-sulfonate (ABTS), 1-ethyl-3-(3-dimethylaminopropyl) carbodiimide (EDC), *N*-hydroxy sulfosuccinimide (NHS), 3-aminopropyltriethoxysilane, and glutaraldehyde were acquired from Sigma-Aldrich Chemicals (St. Louis, MO, USA). Crystal violet (CV), malachite green (MG), and brilliant green (BG) were obtained commercially from Aladdin (Shanghai, China). Graphite flake (100 mesh) was purchased from Nanjing XFNANO Materials Tech Co., Ltd. (Nanjing, China). Ultrapure water (18.2 MΩ cm resistivity) was obtained from an ELGA water purification system (ELGA Berkefeld, Veolia, Germany). All other chemicals were analytical grade and purchased from Sinopharm Chemical Reagent Co., Ltd. (Shanghai, China).

### 3.2. Preparation of Amino Fe_3_O_4_ Nanoparticles

An amount of 1.300 g of ferric chloride, 1.000 g of PEG 6000 and 3.600 g of anhydrous sodium acetate were poured in 40 mL of ethyleneglycol under stirring and ultrasonication. The mixture was then transferred into an autoclave and maintained at 180 °C for 6 h. After reaction, the products were collected by a magnet and washed with ethanol solution three times. Finally, Fe_3_O_4_ nanoparticles were dried in vacuum at 60 °C for further use.

One hundred milligrams of Fe_3_O_4_ nanoparticles were dispersed in 200 mL of 99% ethanol solution under mechanical agitation. Then, 2 mL of 3-aminopropyltriethoxysilane was added dropwise, and the solution was stirred with mechanical agitation at 25 °C for 6 h. The final amino Fe_3_O_4_ nanoparticles were washed with ethanol three times and dried in vacuum at 60 °C.

### 3.3. Preparation of GO

One gram of graphite flakes and 6.000 g KMnO_4_ were slowly added into the mixture containing 120 mL of concentrated sulfuric acid and 13 mL of phosphoric acid. After being maintained at 50 °C for 12 h under magnetic agitation, the mixed solution was poured into 130 mL of ice containing H_2_O_2_. Then, the products were centrifuged at 10,000 rpm for 10 min. The supernatant was decanted, and the solid was washed with 30% hydrochloric acid and water five times, respectively.

### 3.4. Preparation of MGO-Laccase

In order to activate the carboxyl group of GO, 10 mL of EDC/NHS mixture (5 mg/mL of EDC and 3 mg/mL of NHS) was added to 10 mL of GO solution (1 mg/mL in water) and shaken at 25 °C for 30 min. Then, 10 mg of amino Fe_3_O_4_ nanoparticles and 2 mL of laccase were added into the activated GO solution. The mixture was then shaken at 25 °C for 1 h. After reaction, the excess enzyme solution was decanted by magnetic separation. The MGO-laccase were washed with phosphate buffer solution three times and dispersed in PBS for further use.

### 3.5. Characterizations and Measurements

Transmission electron micrographs (TEM) were obtained on a JEM-2100 electron microscope (JEOL, Akishima, Japan). A small amount of sample powder was dispersed in ethanol and dropped on a holey carbon coated copper grid before TEM characterization. Magnetization curves were recorded on a vibration sample magnetometer VSM7307 (Lake Shore, Westerville, Ohio, USA) at room temperature. UV-Vis spectra were recorded on UV-2600 UV-VIS Spectrophotometer (Shimadzu, Kyoto, Japan). In order to investigate the immobilization capacity of the materials, the immobilization amounts of laccase on MGO were performed and calculated by subtracting the amount of enzyme in the supernatant from the amount of total enzyme used for immobilization by Bradford’s method [44]. The retained activity of MGO-laccase is calculated according to Equation (1):

*Retained activity = V_i_/V_f_* × 100% (1)

where V_i_ is the activity of MGO-laccase and V_f_ is the activity of the same amount of free laccase as that immobilized on MGO-laccase [45]. The relative activity of MGO-laccase is expressed as relative forms (%) with the maximal value of activity at a certain concentration set as 100% [46]. Three replications of all assays were conducted in this study.

### 3.6. Laccase Activity Assay

The activities of free laccase and MGO-laccase were determined by a UV-Vis spectrophotometer using ABTS as the substrate. Briefly, 1.0 mL of laccase (5.0 mg/mL, pH 3.0) or MGO-laccase (10 mg) and 0.5 mol/mL of ABTS (2.0 mL, pH 3.0) were mixed and incubated at 30 °C for 20 min. The absorbance of the solutions was measured at 420 nm. The same amount of water instead of enzyme solution was used as a blank test. One laccase activity unit (U) is defined as the amount of enzyme required to oxidize 1 µmol of ABTS per minute. Three replications of all assays were conducted.

To determine the effect of pH on the activity of the enzyme, free laccase and MGO-laccase were respectively incubated in citrate-phosphate buffers, with pH ranging from 2.0 to 8.0 at 25 °C for 15 min, and the activities were then assayed. Meanwhile, the effect of temperature on enzyme activity was determined by incubating free laccase and MGO-laccase in citrate-phosphate buffers (0.1 M, pH 3.0) for 15 min at different temperatures ranging from 5 to 65 °C before activity assays.

### 3.7. Reusability of MGO-Laccase

The reusability of MGO-laccase was evaluated by performing several consecutive operating cycles using ABTS solution as the substrate. After a previous test, the mixture was separated with magnet and the solution was determined by the UV-Vis spectrophotometer. The MGO-laccase was reused by washing excessively with water. Then, the MGO-laccase was transferred into a fresh ABTS solution to start a new cycle. The activity of MGO-laccase in each cycle was measured and shown as relative value. The activity of the first cycle was set as 100%.

### 3.8. Decolorization of Dyes with Free Laccase and MGO-Laccase

The decolorization capacities of various dyes with free laccase and MGO-laccase were conducted, and the decrease in absorbance at the maximum absorption wavelength of each dye (584 nm for CV, 618 nm for MG, and 625 nm for BG) was monitored by a UV-Vis spectrophotometer. One milliliter of laccase (5.0 mg/mL, pH 3.0) or MGO-laccase (10 mg) was mixed with 3.0 mL of dye solution (CV, MG, and BG, 50.0 mg/L) in a citrate-phosphate buffer (0.1 M, pH 3.0). The mixtures were incubated at 35 °C with a shaking speed of 250 rpm in darkness. The blank control used water instead of a free enzyme solution. Experiments were all performed in triplicate. Decolorization capacity was expressed in terms of percentage and calculated as Equation (2):

*Decolorization capacity = (A_i_* − *A*_0_)/*A_i_* × 100% (2)

where A_i_ is the initial absorbance of the dye solution, and A_0_ is the final absorbance of dye after treatment at a certain time.

## 4. Conclusions

In summary, the immobilization of laccase on MGO nanomaterials was successfully achieved. Compared to the free laccase, MGO-laccase showed improved thermal and pH stabilities. At the optimum pH and temperature conditions (pH 3.0 and 35 °C), the MGO-laccase exhibited sufficient magnetic response and satisfactory reusability. The MGO-laccase recovered 59.8% activity after ten uses. MGO-laccase was then utilized in the decolorization of dye solutions and the removal of crystal violet (CV), malachite green (MG), and brilliant green (BG) reached 94.7%, 95.6%, and 91.4% in aqueous solution. The experimental results indicated that the MGO-laccase nanomaterials effectively improved the processing efficiency and expanded the industrial application of enzymes.
